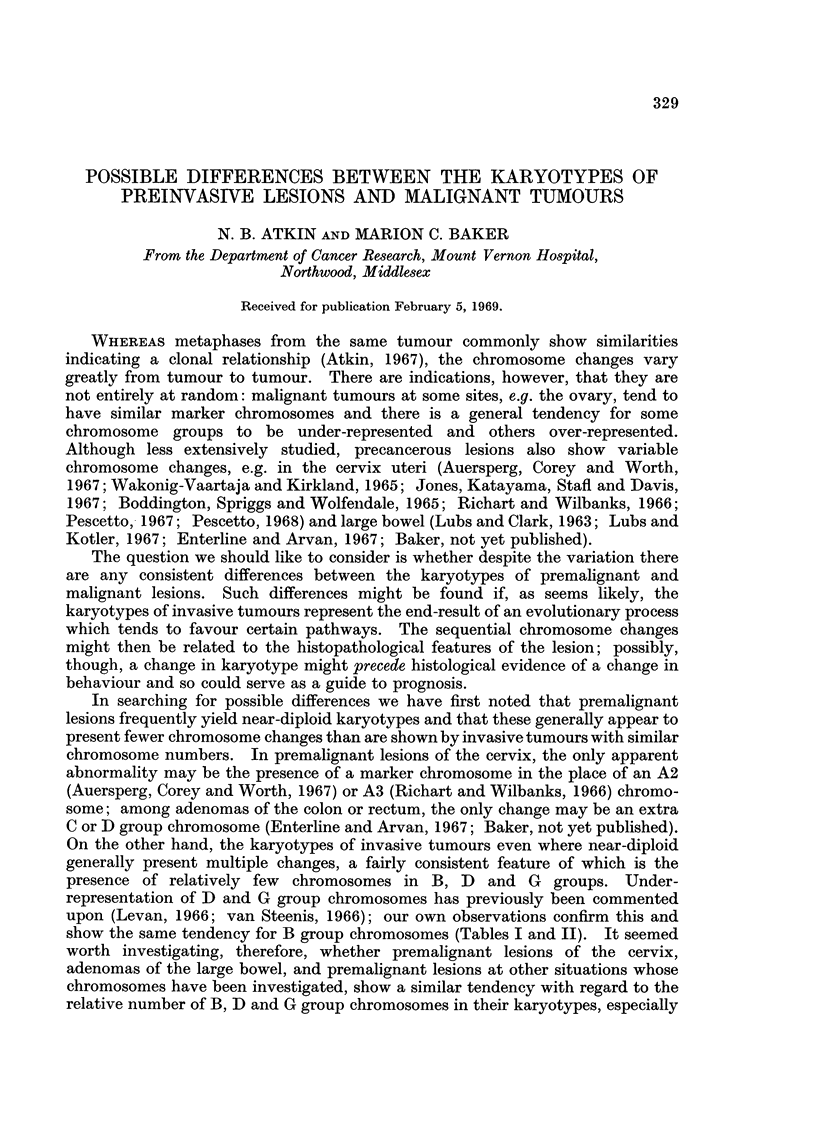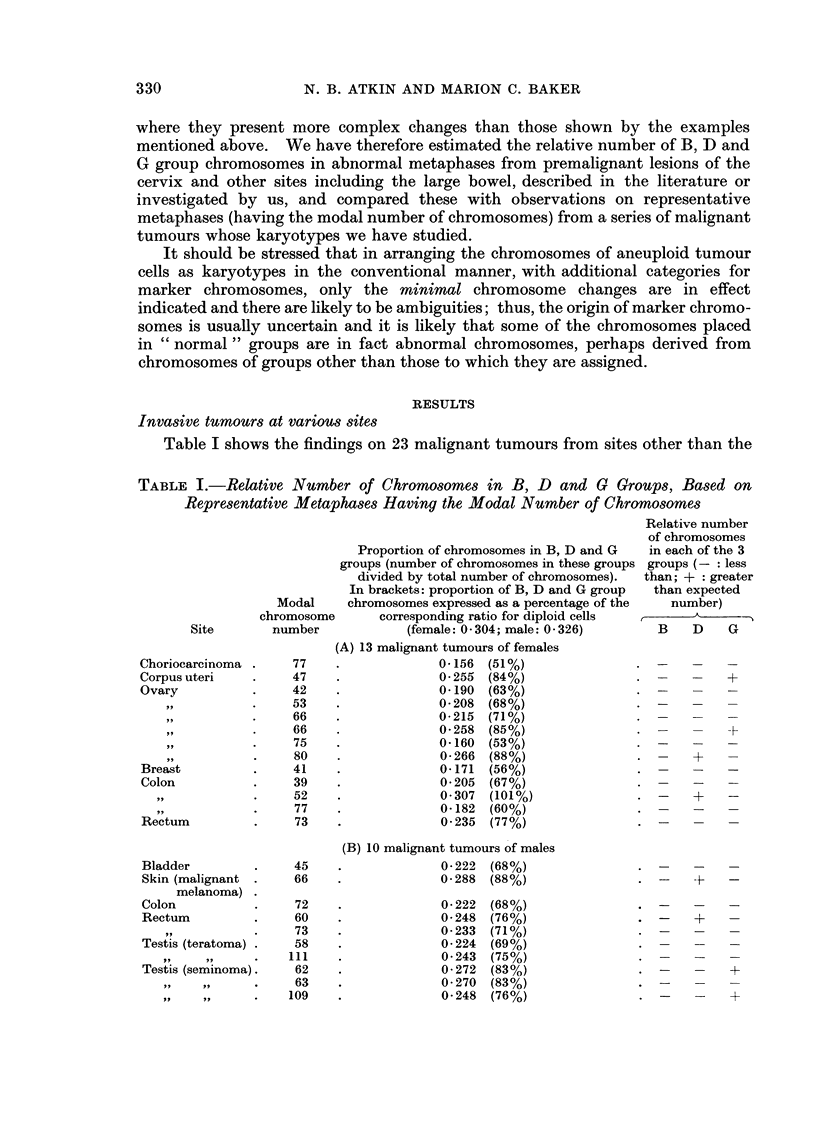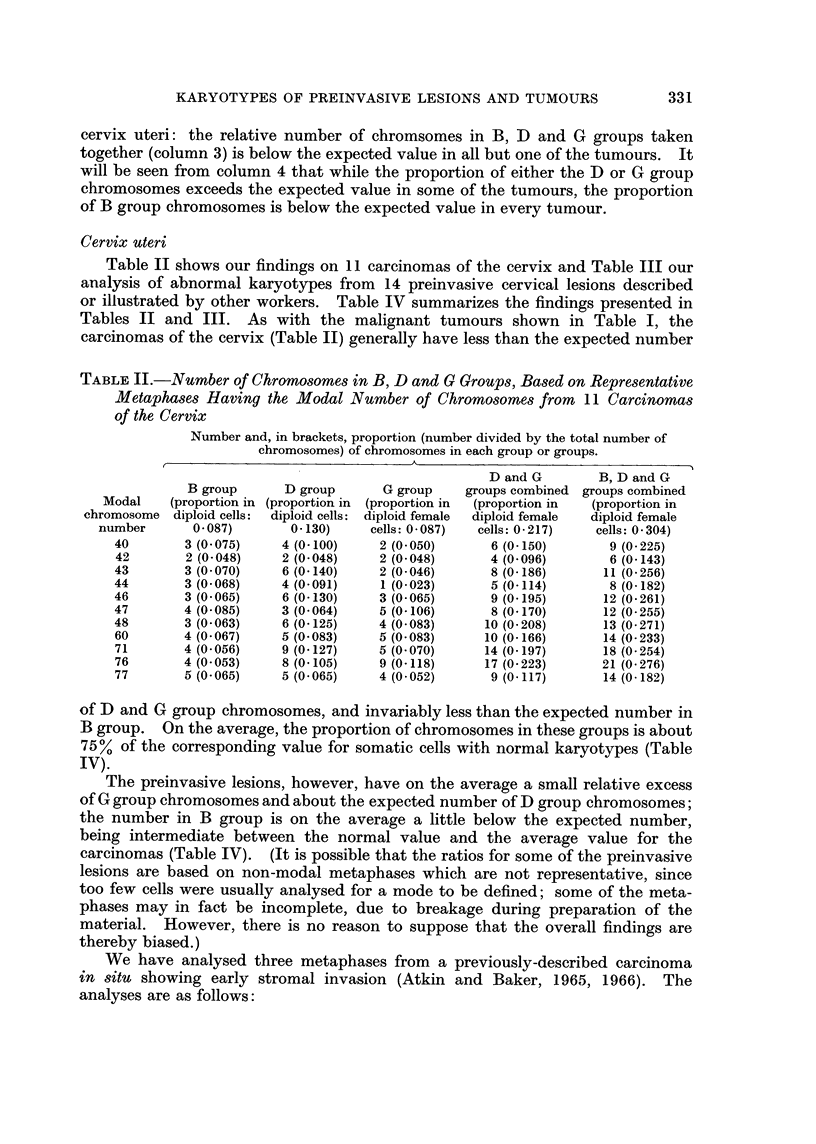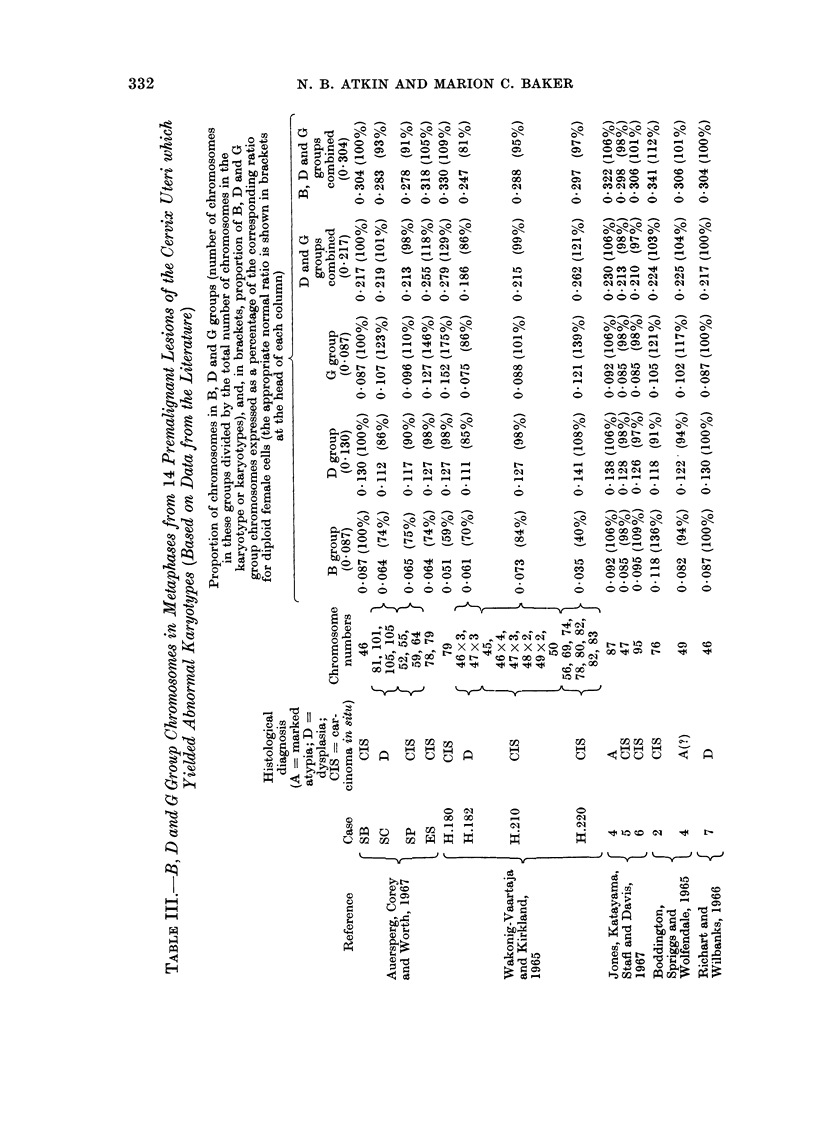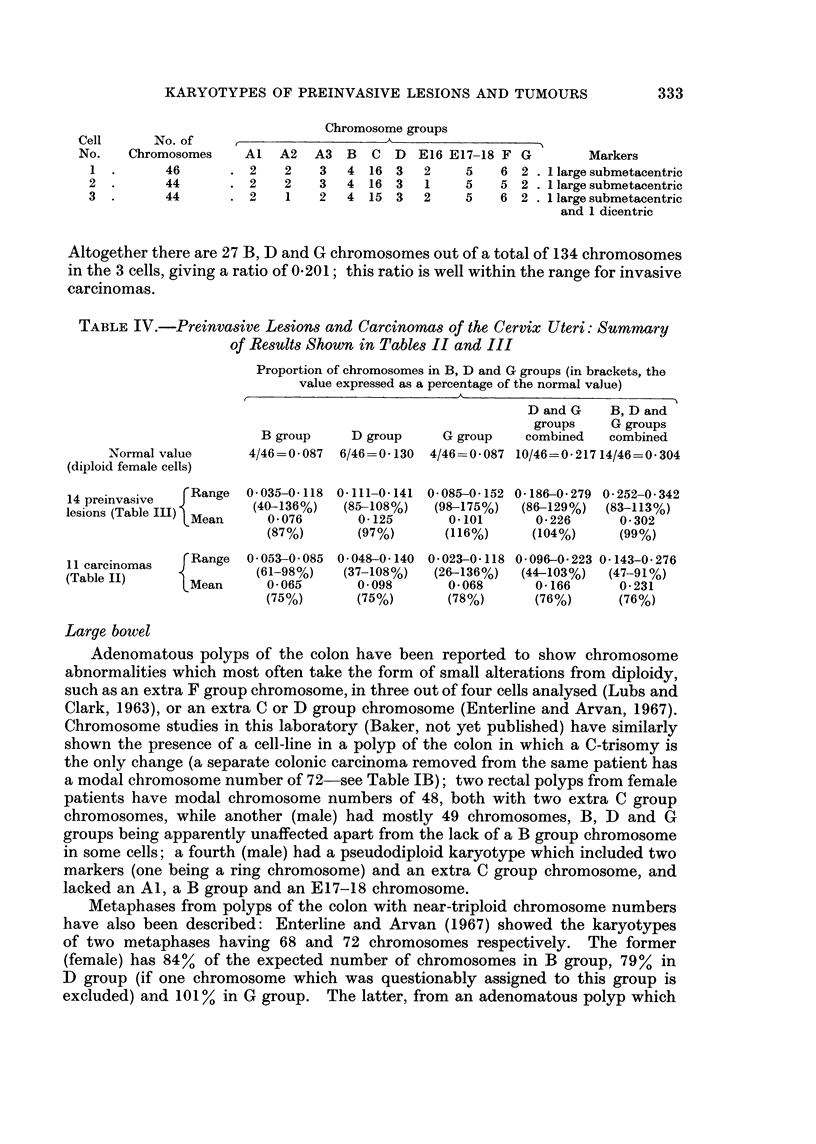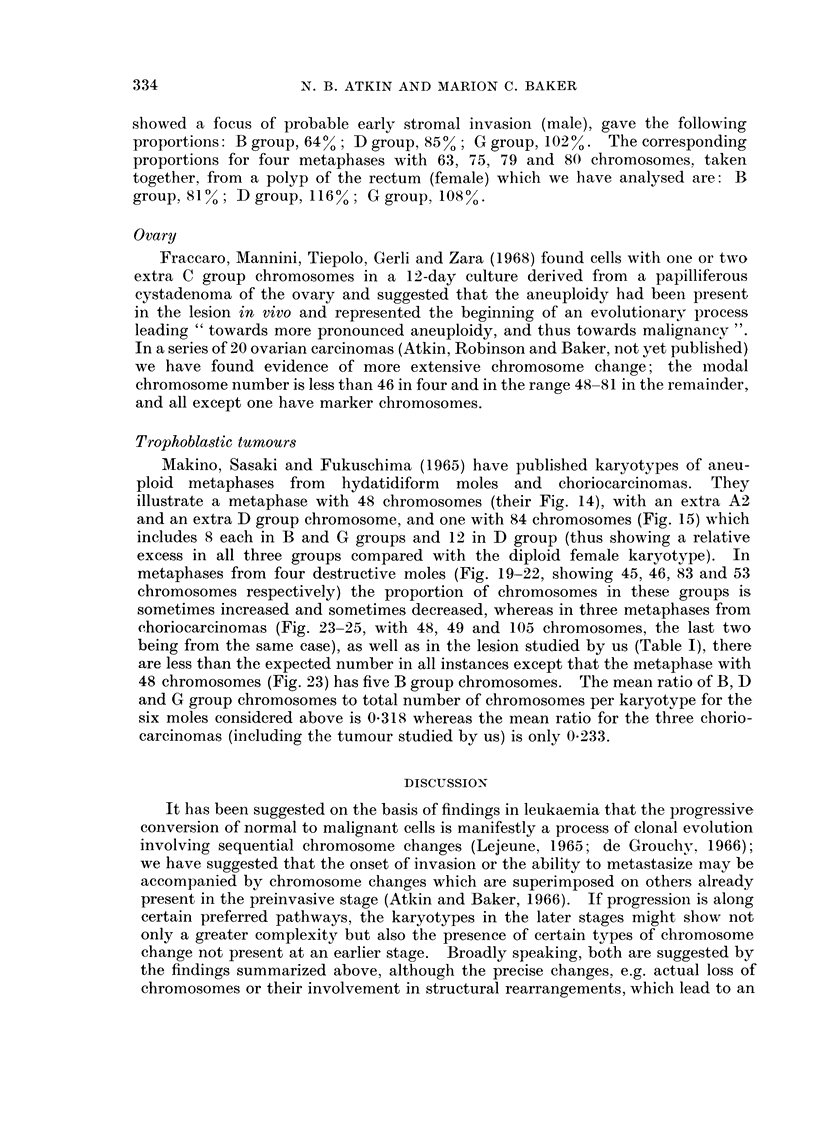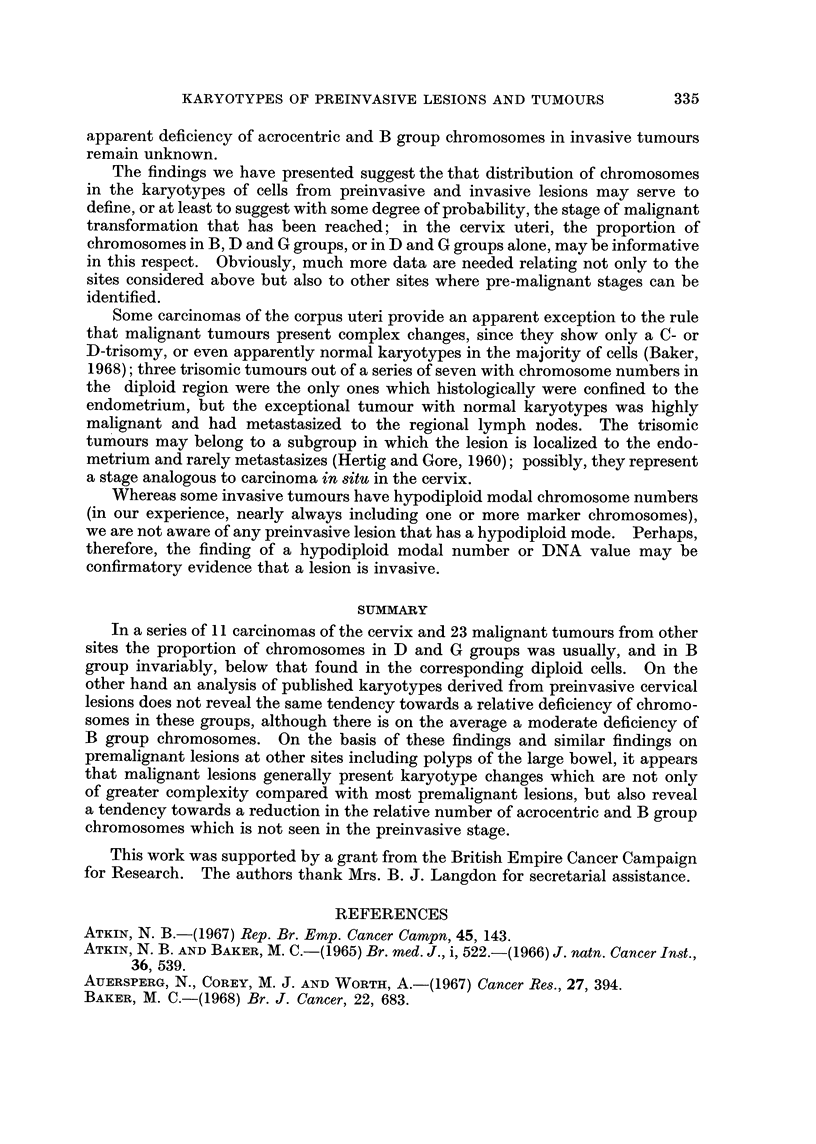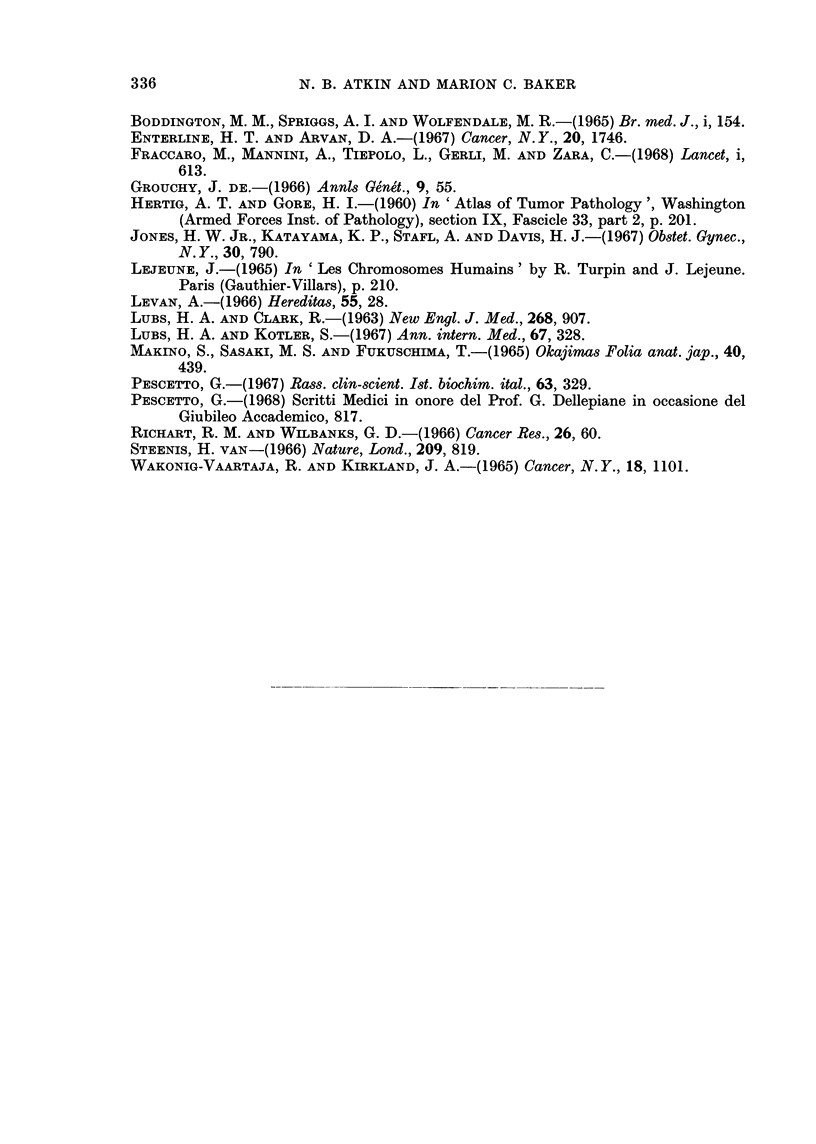# Possible differences between the karyotypes of preinvasive lesions and malignant tumours.

**DOI:** 10.1038/bjc.1969.42

**Published:** 1969-06

**Authors:** N. B. Atkin, M. C. Baker


### POSSIBLE DIFFERENCES BETWEEN THE KARYOTYPES OF PREINVASIVE LESIONS AND MALIGNANT TUMOURS

N. B. ATKIN AND MARION C. BAKER

*From the Department of Cancer Research, Mount Vernon Hospital, Northwood, Middlesex*

Received for publication February 5, 1969.

WHEREAS metaphases from the same tumour commonly show similarities indicating a clonal relationship (Atkin, 1967), the chromosome changes vary greatly from tumour to tumour. There are indications, however, that they are not entirely at random: malignant tumours at some sites, *e.g.* the ovary, tend to have similar marker chromosomes and there is a general tendency for some chromosome groups to be under-represented and others over-represented. Although less extensively studied, precancerous lesions also show variable chromosome changes, e.g. in the cervix uteri (Auersperg, Corey and Worth, 1967; Wakonig-Vaartaja and Kirkland, 1965; Jones, Katayama, Stafl and Davis, 1967; Boddington, Spriggs and Wolfendale, 1965; Richart and Wilbanks, 1966; Pescetto, 1967; Pescetto, 1968) and large bowel (Lubs and Clark, 1963; Lubs and Kotler, 1967; Enterline and Arvan, 1967; Baker, not yet published).

The question we should like to consider is whether despite the variation there are any consistent differences between the karyotypes of premalignant and malignant lesions. Such differences might be found if, as seems likely, the karyotypes of invasive tumours represent the end-result of an evolutionary process which tends to favour certain pathways. The sequential chromosome changes might then be related to the histopathological features of the lesion; possibly, though, a change in karyotype might *precede* histological evidence of a change in behaviour and so could serve as a guide to prognosis.

In searching for possible differences we have first noted that premalignant lesions frequently yield near-diploid karyotypes and that these generally appear to present fewer chromosome changes than are shown by invasive tumours with similar chromosome numbers. In premalignant lesions of the cervix, the only apparent abnormality may be the presence of a marker chromosome in the place of an A2 (Auersperg, Corey and Worth, 1967) or A3 (Richart and Wilbanks, 1966) chromosome; among adenomas of the colon or rectum, the only change may be an extra C or D group chromosome (Enterline and Arvan, 1967; Baker, not yet published). On the other hand, the karyotypes of invasive tumours even where near-diploid generally present multiple changes, a fairly consistent feature of which is the presence of relatively few chromosomes in B, D and G groups. Under-representation of D and G group chromosomes has previously been commented upon (Levan, 1966; van Steenis, 1966); our own observations confirm this and show the same tendency for B group chromosomes (Tables I and II). It seemed worth investigating, therefore, whether premalignant lesions of the cervix, adenomas of the large bowel, and premalignant lesions at other situations whose chromosomes have been investigated, show a similar tendency with regard to the relative number of B, D and G group chromosomes in their karyotypes, especially where they present more complex changes than those shown by the examples mentioned above. We have therefore estimated the relative number of B, D and G group chromosomes in abnormal metaphases from premalignant lesions of the cervix and other sites including the large bowel, described in the literature or investigated by us, and compared these with observations on representative metaphases (having the modal number of chromosomes) from a series of malignant tumours whose karyotypes we have studied.

It should be stressed that in arranging the chromosomes of aneuploid tumour cells as karyotypes in the conventional manner, with additional categories for marker chromosomes, only the *minimal* chromosome changes are in effect indicated and there are likely to be ambiguities; thus, the origin of marker chromosomes is usually uncertain and it is likely that some of the chromosomes placed in " normal " groups are in fact abnormal chromosomes, perhaps derived from chromosomes of groups other than those to which they are assigned.

### RESULTS

### *Invasive tumours at various sites*

Table I shows the findings on 23 malignant tumours from sites other than the

TABLE I.—*Relative Number of Chromosomes in B, D and G Groups, Based on Representative Metaphases Having the Modal Number of Chromosomes*

| Site | Modal chromosome number | Proportion of chromosomes in B, D and G groups (number of chromosomes in these groups divided by total number of chromosomes). In brackets: proportion of B, D and G group chromosomes expressed as a percentage of the corresponding ratio for diploid cells (female: 0·304; male: 0·326) | Relative number of chromosomes in each of the 3 groups (— : less than; + : greater than expected number) | | |
|---|---|---|---|---|---|
| | | | B | D | G |
| | | (A) 13 malignant tumours of females | | | |
| Choriocarcinoma | 77 | 0·156 (51%) | — | — | — |
| Corpus uteri | 47 | 0·255 (84%) | — | — | + |
| Ovary | 42 | 0·190 (63%) | — | — | — |
| ,, | 53 | 0·208 (68%) | — | — | — |
| ,, | 66 | 0·215 (71%) | — | — | — |
| ,, | 66 | 0·258 (85%) | — | — | + |
| ,, | 75 | 0·160 (53%) | — | — | — |
| ,, | 80 | 0·266 (88%) | — | + | — |
| Breast | 41 | 0·171 (56%) | — | — | — |
| Colon | 39 | 0·205 (67%) | — | — | — |
| ,, | 52 | 0·307 (101%) | — | + | — |
| ,, | 77 | 0·182 (60%) | — | — | — |
| Rectum | 73 | 0·235 (77%) | — | — | — |
| | | (B) 10 malignant tumours of males | | | |
| Bladder | 45 | 0·222 (68%) | — | — | — |
| Skin (malignant melanoma) | 66 | 0·288 (88%) | — | + | — |
| Colon | 72 | 0·222 (68%) | — | — | — |
| Rectum | 60 | 0·248 (76%) | — | + | — |
| ,, | 73 | 0·233 (71%) | — | — | — |
| Testis (teratoma) | 58 | 0·224 (69%) | — | — | — |
| ,, ,, | 111 | 0·243 (75%) | — | — | — |
| Testis (seminoma) | 62 | 0·272 (83%) | — | — | + |
| ,, ,, | 63 | 0·270 (83%) | — | — | — |
| ,, ,, | 109 | 0·248 (76%) | — | — | + |

cervix uteri: the relative number of chromsomes in B, D and G groups taken together (column 3) is below the expected value in all but one of the tumours. It will be seen from column 4 that while the proportion of either the D or G group chromosomes exceeds the expected value in some of the tumours, the proportion of B group chromosomes is below the expected value in every tumour.

*Cervix uteri*

Table II shows our findings on 11 carcinomas of the cervix and Table III our analysis of abnormal karyotypes from 14 preinvasive cervical lesions described or illustrated by other workers. Table IV summarizes the findings presented in Tables II and III. As with the malignant tumours shown in Table I, the carcinomas of the cervix (Table II) generally have less than the expected number

TABLE II.—*Number of Chromosomes in B, D and G Groups, Based on Representative Metaphases Having the Modal Number of Chromosomes from 11 Carcinomas of the Cervix*

| | Number and, in brackets, proportion (number divided by the total number of chromosomes) of chromosomes in each group or groups. | | | | |
|---|---|---|---|---|---|
| Modal chromosome number | B group (proportion in diploid cells: 0·087) | D group (proportion in diploid cells: 0·130) | G group (proportion in diploid female cells: 0·087) | D and G groups combined (proportion in diploid female cells: 0·217) | B, D and G groups combined (proportion in diploid female cells: 0·304) |
| 40 | 3 (0·075) | 4 (0·100) | 2 (0·050) | 6 (0·150) | 9 (0·225) |
| 42 | 2 (0·048) | 2 (0·048) | 2 (0·048) | 4 (0·096) | 6 (0·143) |
| 43 | 3 (0·070) | 6 (0·140) | 2 (0·046) | 8 (0·186) | 11 (0·256) |
| 44 | 3 (0·068) | 4 (0·091) | 1 (0·023) | 5 (0·114) | 8 (0·182) |
| 46 | 3 (0·065) | 6 (0·130) | 3 (0·065) | 9 (0·195) | 12 (0·261) |
| 47 | 4 (0·085) | 3 (0·064) | 5 (0·106) | 8 (0·170) | 12 (0·255) |
| 48 | 3 (0·063) | 6 (0·125) | 4 (0·083) | 10 (0·208) | 13 (0·271) |
| 60 | 4 (0·067) | 5 (0·083) | 5 (0·083) | 10 (0·166) | 14 (0·233) |
| 71 | 4 (0·056) | 9 (0·127) | 5 (0·070) | 14 (0·197) | 18 (0·254) |
| 76 | 4 (0·053) | 8 (0·105) | 9 (0·118) | 17 (0·223) | 21 (0·276) |
| 77 | 5 (0·065) | 5 (0·065) | 4 (0·052) | 9 (0·117) | 14 (0·182) |

of D and G group chromosomes, and invariably less than the expected number in B group. On the average, the proportion of chromosomes in these groups is about 75% of the corresponding value for somatic cells with normal karyotypes (Table IV).

The preinvasive lesions, however, have on the average a small relative excess of G group chromosomes and about the expected number of D group chromosomes; the number in B group is on the average a little below the expected number, being intermediate between the normal value and the average value for the carcinomas (Table IV). (It is possible that the ratios for some of the preinvasive lesions are based on non-modal metaphases which are not representative, since too few cells were usually analysed for a mode to be defined; some of the metaphases may in fact be incomplete, due to breakage during preparation of the material. However, there is no reason to suppose that the overall findings are thereby biased.)

We have analysed three metaphases from a previously-described carcinoma *in situ* showing early stromal invasion (Atkin and Baker, 1965, 1966). The analyses are as follows:

TABLE III.—*B, D and G Group Chromosomes in Metaphases from 14 Premalignant Lesions of the Cervix Uteri which Yielded Abnormal Karyotypes (Based on Data from the Literature)*

| Reference | Case | Histological diagnosis (A = marked atypia; D = dysplasia; CIS = carcinoma *in situ*) | Chromosome numbers | Proportion of chromosomes in B, D and G groups (number of chromosomes in these groups divided by the total number of chromosomes in the karyotype or karyotypes), and, in brackets, proportion of B, D and G group chromosomes expressed as a percentage of the corresponding ratio for diploid female cells (the appropriate normal ratio is shown in brackets at the head of each column) | | | | |
|---|---|---|---|---|---|---|---|---|
| | | | | B group (0·087) | D group (0·130) | G group (0·087) | D and G groups combined (0·217) | B, D and G groups combined (0·304) |
| Auersperg, Corey and Worth, 1967 | SB | CIS | 46 | 0·087 (100%) | 0·130 (100%) | 0·087 (100%) | 0·217 (100%) | 0·304 (100%) |
| | SC | D | 81, 101, 105, 105 | 0·064 (74%) | 0·112 (86%) | 0·107 (123%) | 0·219 (101%) | 0·283 (93%) |
| | SP | CIS | 52, 55, 59, 64 | 0·065 (75%) | 0·117 (90%) | 0·096 (110%) | 0·213 (98%) | 0·278 (91%) |
| | ES | CIS | 78, 79 | 0·064 (74%) | 0·127 (98%) | 0·127 (146%) | 0·255 (118%) | 0·318 (105%) |
| Wakonig-Vaartaja and Kirkland, 1965 | H.180 | CIS | 79 | 0·051 (59%) | 0·127 (98%) | 0·152 (175%) | 0·279 (129%) | 0·330 (109%) |
| | H.182 | D | 46×3, 47×3 | 0·061 (70%) | 0·111 (85%) | 0·075 (86%) | 0·186 (86%) | 0·247 (81%) |
| | H.210 | CIS | 45, 46×4, 47×3, 48×2, 49×2, 50 | 0·073 (84%) | 0·127 (98%) | 0·088 (101%) | 0·215 (99%) | 0·288 (95%) |
| | H.220 | CIS | 56, 69, 74, 78, 80, 82, 82, 83 | 0·035 (40%) | 0·141 (108%) | 0·121 (139%) | 0·262 (121%) | 0·297 (97%) |
| Jones, Katayama, Stafl and Davis, 1967 | 4 | A | 87 | 0·092 (106%) | 0·138 (106%) | 0·092 (106%) | 0·230 (106%) | 0·322 (106%) |
| | 5 | CIS | 47 | 0·085 (98%) | 0·128 (98%) | 0·085 (98%) | 0·213 (98%) | 0·298 (98%) |
| | 6 | CIS | 95 | 0·095 (109%) | 0·126 (97%) | 0·085 (98%) | 0·210 (97%) | 0·306 (101%) |
| Boddington, Spriggs and Wolfendale, 1965 | 2 | CIS | 76 | 0·118 (136%) | 0·118 (91%) | 0·105 (121%) | 0·224 (103%) | 0·341 (112%) |
| | 4 | A(?) | 49 | 0·082 (94%) | 0·122 (94%) | 0·102 (117%) | 0·225 (104%) | 0·306 (101%) |
| Richart and Wilbanks, 1966 | 7 | D | 46 | 0·087 (100%) | 0·130 (100%) | 0·087 (100%) | 0·217 (100%) | 0·304 (100%) |

| Cell No. | No. of Chromosomes | A1 | A2 | A3 | B | C | D | E16 | E17–18 | F | G | Markers |
|---|---|---|---|---|---|---|---|---|---|---|---|---|
| | | Chromosome groups | | | | | | | | | | |
| 1 | 46 | 2 | 2 | 3 | 4 | 16 | 3 | 2 | 5 | 6 | 2 | 1 large submetacentric |
| 2 | 44 | 2 | 2 | 3 | 4 | 16 | 3 | 1 | 5 | 5 | 2 | 1 large submetacentric |
| 3 | 44 | 2 | 1 | 2 | 4 | 15 | 3 | 2 | 5 | 6 | 2 | 1 large submetacentric and 1 dicentric |

Altogether there are 27 B, D and G chromosomes out of a total of 134 chromosomes in the 3 cells, giving a ratio of 0·201; this ratio is well within the range for invasive carcinomas.

TABLE IV.—*Preinvasive Lesions and Carcinomas of the Cervix Uteri: Summary of Results Shown in Tables II and III*

| | | Proportion of chromosomes in B, D and G groups (in brackets, the value expressed as a percentage of the normal value) | | | | |
|---|---|---|---|---|---|---|
| | | B group | D group | G group | D and G groups combined | B, D and G groups combined |
| Normal value (diploid female cells) | | 4/46=0·087 | 6/46=0·130 | 4/46=0·087 | 10/46=0·217 | 14/46=0·304 |
| 14 preinvasive lesions (Table III) | Range | 0·035–0·118 (40–136%) | 0·111–0·141 (85–108%) | 0·085–0·152 (98–175%) | 0·186–0·279 (86–129%) | 0·252–0·342 (83–113%) |
| | Mean | 0·076 (87%) | 0·125 (97%) | 0·101 (116%) | 0·226 (104%) | 0·302 (99%) |
| 11 carcinomas (Table II) | Range | 0·053–0·085 (61–98%) | 0·048–0·140 (37–108%) | 0·023–0·118 (26–136%) | 0·096–0·223 (44–103%) | 0·143–0·276 (47–91%) |
| | Mean | 0·065 (75%) | 0·098 (75%) | 0·068 (78%) | 0·166 (76%) | 0·231 (76%) |

*Large bowel*

Adenomatous polyps of the colon have been reported to show chromosome abnormalities which most often take the form of small alterations from diploidy, such as an extra F group chromosome, in three out of four cells analysed (Lubs and Clark, 1963), or an extra C or D group chromosome (Enterline and Arvan, 1967). Chromosome studies in this laboratory (Baker, not yet published) have similarly shown the presence of a cell-line in a polyp of the colon in which a C-trisomy is the only change (a separate colonic carcinoma removed from the same patient has a modal chromosome number of 72—see Table IB); two rectal polyps from female patients have modal chromosome numbers of 48, both with two extra C group chromosomes, while another (male) had mostly 49 chromosomes, B, D and G groups being apparently unaffected apart from the lack of a B group chromosome in some cells; a fourth (male) had a pseudodiploid karyotype which included two markers (one being a ring chromosome) and an extra C group chromosome, and lacked an A1, a B group and an E17–18 chromosome.

Metaphases from polyps of the colon with near-triploid chromosome numbers have also been described: Enterline and Arvan (1967) showed the karyotypes of two metaphases having 68 and 72 chromosomes respectively. The former (female) has 84% of the expected number of chromosomes in B group, 79% in D group (if one chromosome which was questionably assigned to this group is excluded) and 101% in G group. The latter, from an adenomatous polyp which showed a focus of probable early stromal invasion (male), gave the following proportions: B group, 64%; D group, 85%; G group, 102%. The corresponding proportions for four metaphases with 63, 75, 79 and 80 chromosomes, taken together, from a polyp of the rectum (female) which we have analysed are: B group, 81%; D group, 116%; G group, 108%.

*Ovary*

Fraccaro, Mannini, Tiepolo, Gerli and Zara (1968) found cells with one or two extra C group chromosomes in a 12-day culture derived from a papilliferous cystadenoma of the ovary and suggested that the aneuploidy had been present in the lesion *in vivo* and represented the beginning of an evolutionary process leading " towards more pronounced aneuploidy, and thus towards malignancy ". In a series of 20 ovarian carcinomas (Atkin, Robinson and Baker, not yet published) we have found evidence of more extensive chromosome change; the modal chromosome number is less than 46 in four and in the range 48–81 in the remainder, and all except one have marker chromosomes.

*Trophoblastic tumours*

Makino, Sasaki and Fukuschima (1965) have published karyotypes of aneuploid metaphases from hydatidiform moles and choriocarcinomas. They illustrate a metaphase with 48 chromosomes (their Fig. 14), with an extra A2 and an extra D group chromosome, and one with 84 chromosomes (Fig. 15) which includes 8 each in B and G groups and 12 in D group (thus showing a relative excess in all three groups compared with the diploid female karyotype). In metaphases from four destructive moles (Fig. 19–22, showing 45, 46, 83 and 53 chromosomes respectively) the proportion of chromosomes in these groups is sometimes increased and sometimes decreased, whereas in three metaphases from choriocarcinomas (Fig. 23–25, with 48, 49 and 105 chromosomes, the last two being from the same case), as well as in the lesion studied by us (Table I), there are less than the expected number in all instances except that the metaphase with 48 chromosomes (Fig. 23) has five B group chromosomes. The mean ratio of B, D and G group chromosomes to total number of chromosomes per karyotype for the six moles considered above is 0·318 whereas the mean ratio for the three choriocarcinomas (including the tumour studied by us) is only 0·233.

### DISCUSSION

It has been suggested on the basis of findings in leukaemia that the progressive conversion of normal to malignant cells is manifestly a process of clonal evolution involving sequential chromosome changes (Lejeune, 1965; de Grouchy, 1966); we have suggested that the onset of invasion or the ability to metastasize may be accompanied by chromosome changes which are superimposed on others already present in the preinvasive stage (Atkin and Baker, 1966). If progression is along certain preferred pathways, the karyotypes in the later stages might show not only a greater complexity but also the presence of certain types of chromosome change not present at an earlier stage. Broadly speaking, both are suggested by the findings summarized above, although the precise changes, e.g. actual loss of chromosomes or their involvement in structural rearrangements, which lead to an apparent deficiency of acrocentric and B group chromosomes in invasive tumours remain unknown.

The findings we have presented suggest the that distribution of chromosomes in the karyotypes of cells from preinvasive and invasive lesions may serve to define, or at least to suggest with some degree of probability, the stage of malignant transformation that has been reached; in the cervix uteri, the proportion of chromosomes in B, D and G groups, or in D and G groups alone, may be informative in this respect. Obviously, much more data are needed relating not only to the sites considered above but also to other sites where pre-malignant stages can be identified.

Some carcinomas of the corpus uteri provide an apparent exception to the rule that malignant tumours present complex changes, since they show only a C- or D-trisomy, or even apparently normal karyotypes in the majority of cells (Baker, 1968); three trisomic tumours out of a series of seven with chromosome numbers in the diploid region were the only ones which histologically were confined to the endometrium, but the exceptional tumour with normal karyotypes was highly malignant and had metastasized to the regional lymph nodes. The trisomic tumours may belong to a subgroup in which the lesion is localized to the endometrium and rarely metastasizes (Hertig and Gore, 1960); possibly, they represent a stage analogous to carcinoma *in situ* in the cervix.

Whereas some invasive tumours have hypodiploid modal chromosome numbers (in our experience, nearly always including one or more marker chromosomes), we are not aware of any preinvasive lesion that has a hypodiploid mode. Perhaps, therefore, the finding of a hypodiploid modal number or DNA value may be confirmatory evidence that a lesion is invasive.

### SUMMARY

In a series of 11 carcinomas of the cervix and 23 malignant tumours from other sites the proportion of chromosomes in D and G groups was usually, and in B group invariably, below that found in the corresponding diploid cells. On the other hand an analysis of published karyotypes derived from preinvasive cervical lesions does not reveal the same tendency towards a relative deficiency of chromosomes in these groups, although there is on the average a moderate deficiency of B group chromosomes. On the basis of these findings and similar findings on premalignant lesions at other sites including polyps of the large bowel, it appears that malignant lesions generally present karyotype changes which are not only of greater complexity compared with most premalignant lesions, but also reveal a tendency towards a reduction in the relative number of acrocentric and B group chromosomes which is not seen in the preinvasive stage.

This work was supported by a grant from the British Empire Cancer Campaign for Research. The authors thank Mrs. B. J. Langdon for secretarial assistance.

### REFERENCES

Atkin, N. B.—(1967) *Rep. Br. Emp. Cancer Campn*, **45**, 143.
Atkin, N. B. and Baker, M. C.—(1965) *Br. med. J.*, i, 522.—(1966) *J. natn. Cancer Inst.*, **36**, 539.
Auersperg, N., Corey, M. J. and Worth, A.—(1967) *Cancer Res.*, **27**, 394.
Baker, M. C.—(1968) *Br. J. Cancer*, 22, 683.

BODDINGTON, M. M., SPRIGGS, A. I. AND WOLFENDALE, M. R.—(1965) *Br. med. J.*, i, 154.
ENTERLINE, H. T. AND ARVAN, D. A.—(1967) *Cancer, N.Y.*, **20**, 1746.
FRACCARO, M., MANNINI, A., TIEPOLO, L., GERLI, M. AND ZARA, C.—(1968) *Lancet*, i, 613.
GROUCHY, J. DE.—(1966) *Annls Génét.*, **9**, 55.
HERTIG, A. T. AND GORE, H. I.—(1960) *In* ' Atlas of Tumor Pathology ', Washington (Armed Forces Inst. of Pathology), section IX, Fascicle 33, part 2, p. 201.
JONES, H. W. JR., KATAYAMA, K. P., STAFL, A. AND DAVIS, H. J.—(1967) *Obstet. Gynec., N.Y.*, **30**, 790.
LEJEUNE, J.—(1965) *In* ' Les Chromosomes Humains ' by R. Turpin and J. Lejeune. Paris (Gauthier-Villars), p. 210.
LEVAN, A.—(1966) *Hereditas*, **55**, 28.
LUBS, H. A. AND CLARK, R.—(1963) *New Engl. J. Med.*, **268**, 907.
LUBS, H. A. AND KOTLER, S.—(1967) *Ann. intern. Med.*, **67**, 328.
MAKINO, S., SASAKI, M. S. AND FUKUSCHIMA, T.—(1965) *Okajimas Folia anat. jap.*, **40**, 439.
PESCETTO, G.—(1967) *Rass. clin-scient. Ist. biochim. ital.*, **63**, 329.
PESCETTO, G.—(1968) Scritti Medici in onore del Prof. G. Dellepiane in occasione del Giubileo Accademico, 817.
RICHART, R. M. AND WILBANKS, G. D.—(1966) *Cancer Res.*, **26**, 60.
STEENIS, H. VAN—(1966) *Nature, Lond.*, **209**, 819.
WAKONIG-VAARTAJA, R. AND KIRKLAND, J. A.—(1965) *Cancer, N.Y.*, **18**, 1101.